# Seroprevalence of EV-A71 neutralizing antibodies following the 2011 epidemic in HCMC, Vietnam

**DOI:** 10.1371/journal.pntd.0008124

**Published:** 2020-03-03

**Authors:** Fang-Lin Kuo, Truong Huu Khanh, Wan-Yu Chung, Nguyen Thanh Hung, Shu-Ting Luo, Wen-Chiung Chang, Le Nguyen Thanh Nhan, Le Quoc Thinh, Min-Shi Lee

**Affiliations:** 1 National Institute of Infectious Diseases and Vaccinology, National Health Research Institutes, Miaoli, Taiwan; 2 Children’s Hospital No. 1, Ho Chi Minh City, Vietnam; University of Texas Medical Branch, UNITED STATES

## Abstract

Enterovirus-A71 (EV-A71) cyclically causes hand-foot-mouth disease (HFMD) epidemics in Asian children. An EV-A71 epidemic occurred in Southern Vietnam in 2011, but its scale is not clear. We collected residual sera from non-HFMD Vietnamese inpatients in 2012–2013 to determine seroprevalence of EV-A71 neutralizing antibodies, and measured cross-reactive neutralizing antibody titers against three EV-A71 genogroups. About 23.5% of 1-year-old children in Southern Vietnam has been infected by EV-A71, and the median age of infection was estimated to be 3 years. No significant antigenic variation could be detected among the three EV-A71 genogroups. The high seroprevalence of EV-A71 neutralizing antibody in children living in southern Vietnam indicates the necessity of introducing EV-A71 vaccines in southern Vietnam, particularly for children under 6 months of age. Moreover, it is critical to understand EV-A71 disease burden for formulating national vaccination policy.

## Author summary

Enterovirus-A71 (EV-A71), a member of the enterovirus genus, is a virulent pathogen causing neurological complications. EV-A71 mainly spreads through oral-fecal or oral-oral transmission, as well as respiratory droplets. EV-A71 outbreaks have cyclically occurred throughout some Asian countries since 1997, with millions of people affected. The presence of serum neutralizing antibodies to EV-A71 can represent the prevalence of previous EV-A71 infections and seroprevalence studies are widely used to understand prevalence of infectious diseases. The results of our study demonstrate that about 50% of young children under 3 years of age were infected during the 2011 epidemic in southern Vietnam. The high seroprevalence of EV-A71 neutralizing antibody in children living in southern Vietnam indicates the necessity of introducing EV-A71 vaccines, particularly for children under 6 months of age. Moreover, it is critical to understand EV-A71 disease burden for formulating national vaccination policy in the future.

## Introduction

Enterovirus-A71 (EV-A71), a member of the Picornaviridae, is a non-enveloped, single-stranded, positive-sense RNA virus and was first isolated in 1969 in California, USA [1,2]. EV-A71 could be classified into 3 major genogroup (A, B and C) [3] and newly discovered genogroups (D, E and F) [4]. Genogroup A includes the prototype EV-A71 first isolated in California. Genogroup B has five genotypes (B1~B5) which have been circulating in Asia [3]. Genogroup C also comprises five genotypes (C1~C5) and C3—C5 recently have been involved in epidemics in Asia and Europe. Additionally, genotype C4 could be further classified as subgenotype C4a and C4b [5].

In general, EV-A71 is a major cause of HFMD and neurological complications such as aseptic meningitis and encephalomyelitis [3]. In addition, it could lead to central nervous system (CNS) infection without HFMD manifestation. EV-A71 might occasionally cause herpangina although the major causative agent of herpangina is coxsackieviruses [5]. Enterovirus remains prevalent in the Asia-Pacific region where large populations were infected every year with EV-A71 particularly as a major cause of neurological complications and mortality. Since 1997, cyclical EV-A71 outbreaks have occurred in Asian countries including Brunei, Cambodia, China, Malaysia, Singapore, Taiwan, Thailand, and Vietnam [5]. EV-A71 exhibits a high degree of genetic diversity which emphasizes the importance of epidemiological monitoring in the Asia-Pacific region [5]. In Philippines, the HFMD surveillance demonstrated the positive rate of EV-A71 is between 2.0–5.4% with genotype C2 circulating from 2000 to 2016 [6]. In China, C4a represented the most prevalent genotype of EV-A71 infection between 2010–2012 [7]. Genotype/subgenotype B4, C1, C2, C4b, and C5 were found in Thailand in 2000–2009, and the predominant genotype shifted to B5 and C4a in 2011–2017 [8]. To harmonize the enterovirus surveillance in the Asia-Pacific region, the Asia-Pacific Network for Enterovirus Surveillance (APNES) was established in 2017. In 2018, APNES has reported that EV-A71 continued circulating in the Asia-Pacific region where genotype B5 (Sarawak, Malaysia) and C4 (Thailand) have been detected [5,9].

In Vietnam, EV-A71 was first identified in 2003 [10] and large-scale epidemics occurred in 2005, 2011 and 2018, respectively [10–15], and over 53,000 hospitalized and 6 fatal cases were reported in the latest outbreak [13]. Genotype C5 and subgenotype C4a viruses were predominant in the 2005 and 2011 EV-A71 epidemics, respectively, and sporadic C5 viruses were also detected in the 2011 epidemic [11,12]. Based on phylogenetic analyses, the C4a viruses isolated in 2011 were closely related to two C4a lineages in China, and the C5 viruses were genetically similar with that circulating in Vietnam since 2003 [11]. The widespread EV-A71 infections in 2011 in southern Vietnam resulted in huge disease burden. The study conducted in the largest children hospital in Ho Chi Minh City (HCMC) identified 443 severe cases with a grade of 2b or higher among 3,791 HFMD patients [16], and the report system in Vietnam indicated that a total of 170 cases died from HFMD in 2011 [12]. After the 2011 epidemic, EV-A71 genotypes in southern Vietnam switched from C4 in 2011 to B5 in early 2013 [14,15]. Several studies have elucidated virological characteristics of the 2011 EV-A71 epidemic in Vietnam [10–15], yet seroprevalence data are limited. Therefore, we collected residual sera from 562 non-HFMD inpatients admitted to Children Hospital No. 1 (CH1), HCMC from 2012 to 2013, to determine the seroprevalence of EV-A71 neutralizing antibodies.

## Materials and methods

### Ethics statement

The samples were collected as part of a hospital-based surveillance of enterovirus infections. Ethical approval was given by the Institutional Review Boards (Children’s Hospital 1 ethical committee, reference IORG-0007285, FWA00009748). Written consent was obtained from parents/guardians of participating children.

### Study population

CH1 is a pediatric hospital providing health care for children up to 16 years old in HCMC and southern provinces of Vietnam. Residual sera were collected from inpatients who were admitted to CH1 due to illnesses not related to HFMD and herpangina from April 2012 to October 2013. Non-HFMD/herpangina inpatients admitted to CH1 during this period were invited to participate in this study, and those who or whose parents/guardians had signed the informed consent form were recruited. After obtaining the informed consent from the guardians, residual sera were collected from the participating inpatients. A total of 562 subjects were enrolled in the study, and 9 (1.6%) subjects were excluded due to missing data. Thus, 553 subjects with complete data were analyzed.

### Laboratory analysis

Serum neutralizing antibody against subgenotype C4a virus (viral strain: C4a/HCM82/VNM/11, accession code: KC222964) was measured to determine status of EV-A71 infection following standard procedures. RD cells and 100 TCID50 of virus titer were employed for measuring neutralizing antibody titers. Each serum was tested with three replicates. The highest serum dilution which could inhibit more than 50% of wells with cytopathological effect observed under microscope was assigned as neutralizing antibody titer. The procedure of neutralizing antibody tests was described previously [11]. For interpreting cross-reactive serum neutralizing antibody responses, it is better to select children who are only infected by one EV-A71 genotype. The predominant EV-A71 genotype/subgenotype in HCMC was C4 in 2010–2012 and shifted to B5 in early 2013. Therefore, cross-reactive neutralizing antibody titers against three EV-A71 viruses (A/BrCr/1970, B5/HCMC-E452/2013 and C4a/HCM82/VNM/11) were measured using sera collected from seropositive children aged 6 to 24 months. These seropositive children were very likely only infected by EV-A71 C4a viruses during 2011 and 2012.

### Data analysis

Comparisons of seroprevalences across several Asian countries have been conducted. The median ages of infection are assumed as 50% seroprevalence and estimated using the Reed-Muench method. The geometric mean titers (GMT) of antibody titers and 95% confidence intervals (CI) were calculated. Paired t-tests were performed for comparing cross-reactive neutralizing antibody titers because we measured the serum neutralizing antibody titers against the three EV-A71 viruses (A/BrCr/1970, B5/HCMC-E452/2013 and C4a/HCM82/VNM/11) for each child.

## Results

Among the 553 subjects, 350 (63.3%) were males and 203 (37.7%) were females and age-specific seroprevalence of EV-A71 neutralizing antibody were not significantly different between these two gender groups (all P-values >0.05, see S1 Table). As shown in Table 1, the age-specific seroprevalences of EV-A71 neutralizing antibody were 14.9% at under 0.5 year, 17.2% at 0.5–0.9 year, 23.5% at 1–1.9 years, 29.4% at 2–2.9 years, 57.6% at 3–3.9 years, 62.3% at 4–4.9 years, 66.1% at 5–5.9 years, 76.5% at 6–6.9 years, 68.6% at 7–7.9 years, 85.7% at 8–8.9 years and 75.5% at over 9 years. The median age of infection (50% seroprevalence) was about 3 years in southern Vietnam, which is much lower than that in Singapore (>17 years) and Taiwan (>15 years) but is higher than that in Cambodia (1.5 years) and similar to China (3.1 years) (Table 1) [17–21]. In another study collecting sera after the 2005 epidemic in HCMC, seroprevalences of EV-A71 antibody were 5.6% at 1 years of age and 12.2% at 2 years of age [22], which indicate that more young children under 3 years of age were infected in the 2011 epidemic (15.8% at 1 years of age; 23.5% at 2 years of age) than in the 2005 epidemic (S2 Table) [22–25]. In contrast, seroprevalences of EV-A71 antibody in Singapore and Taiwan were much lower in recent years [17,19,23,25]. In addition to the recent seroprevalence data compared in Table 1, several historical seroprevalence studies conducted in Asian countries have also been summarized in S2 Table. It is hard to compare different seroprevalence studies using different serological assays and sampling methods so these data should be interpreted with caution.

**Table 1:** Seroprevalence of EV71 Neutralizing Antibody in HCM City, Vietnam, 2012–2013 (n = 553), compared with some Asian countries.

Age (months)	This study, 2012–2013 n/N (%, 95%CI)	Singapore 2008–2010 [17]	Cambodia 2006–2011 [18]	Taiwan 2017 [19]	Thailand, 2013 [20]	Jiangsu, China, 2010 [21]
Serum collection	non-HFMD inpatients	non-HFMD inpatients	Dengue inpatients	Healthy children in schools	non-HFMD patients	Healthy children in community
<6	7/47 (14.9, 6.2–28.3)				40%	27.8%
6–11	5/29 (17.2, 5.9–35.8)			10% (<1y)	5%	7.5%
12–23	12/51 (23.5, 12.8–37.5)	15.5% (1-6y)		4% (1y)	13%	20%
24–35	15/51 (29.4, 17.5–43.8)		92%	8% (2y)	40%	43%
36–47	34/59 (57.6, 44.1–70.4)		92%	8% (3-5y)	47%	75%
48–59	33/53 (62.3, 47.9–75.2)		95%		65%	85%
60–71	39/59 (66.1, 52.6–77.9)		94%		77% (5-6y)86% (7-11y)	80%
72–83	39/51 (76.5, 62.5–87.2)	26.2% (7-12y)37.1% (13-17y)		31% (6-11y)45% (12-15y)		95%
84–95	35/51 (68.6, 54.1–80.9)					
96–107	42/49 (85.7, 72.8–94.1)					
>108	40/53 (75.5, 61.7–86.2)					
Median (years)*	3	>17	1.5	>15	4.2	3.1

* Median age of infection is defined as 50% seroprevalence and estimated using the Reed-Muench method assuming a linear relationship between age and seroprevalences which cross-over 50%. For the Cambodia study, it is estimated assuming 0% (no natural infection) at birth and 92% at 30 months of age.

**Fig 1:**
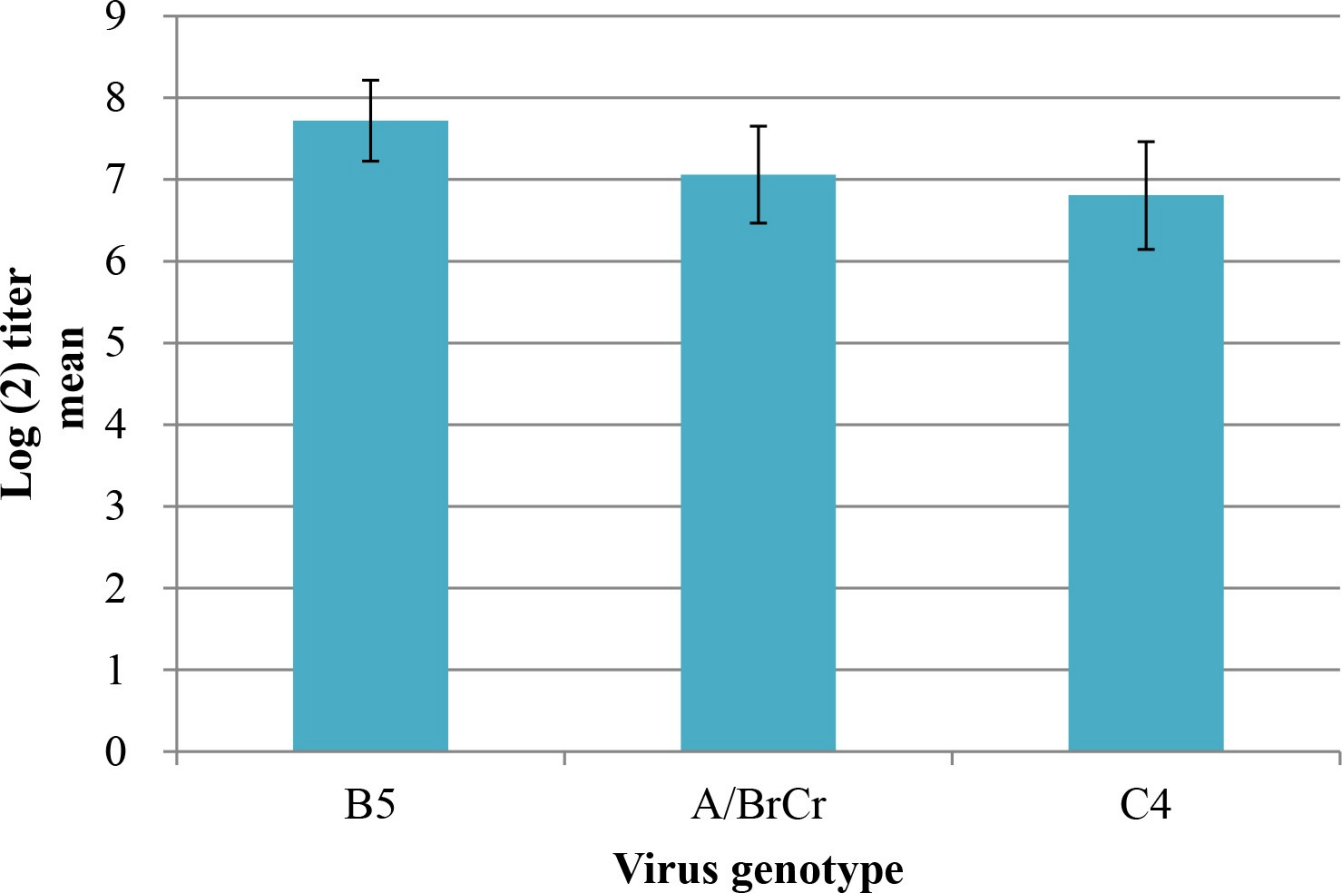
Cross-reactive neutralizing antibody titers in post-infection children sera against EV71 viruses isolated in Vietnam.

It is important to monitor antigenic variation among different EV-A71 genogroups for vaccine development. Therefore, we further selected 33 sera collected from seropositive children aged 6 to 24 months to measure cross-reactive neutralizing antibody titers against three EV-A71 viruses (A/BrCr/1970, B5/HCMC-E452/2013 and C4a/HCM82/VNM/11). Since subgenotype C4a viruses predominantly circulated in 2011 and 2012, these seropositive young children were likely infected with the subgenotype C4a viruses. The geometric mean titers (GMT) of antibody titers and 95% confidence intervals (CI) were calculated. As shown in Fig 1, GMT (95% CI) of neutralizing antibodies against the B5, A and C4a viruses were 7.72 (95%CI = 7.22–8.21), 7.06 (95% CI: 6.47–7.66), and 6.81 (95% CI: 6.15–7.47) after log_2_ transformation, respectively (Fig 1). Overall, no significant antigenic variation could be detected among these three viruses using post-infection sera collected from children infected with subgenotype C4a viruses.

## Discussion

Multiple EV-A71-related fatal epidemics have occurred in southern Vietnam. In this study, we found high seroprevalence of EV-A71 neutralizing antibody in children living in southern Vietnam after the 2011 epidemic, which indicates the necessity of introducing EV-A71 vaccines in southern Vietnam. Moreover, about 17% of children in southern Vietnam were infected at 6–12 months of age during the 2011 epidemic and this age group has the highest risk of developing EV-A71-related neurological complications [23]. Therefore, vaccine development in southern Vietnam should target children under 6 months of age.

Two major EV-A71 genotypes (B5 and C4) are circulating in Asia. The genotype C1, C4, and C5 were reported to be circulating in Vietnam since 2005 and the dominant genotype of EV-A71 shifted from C5 to C4 in 2011 [14,26] and from C4 to B5 in early 2013 [14]. In this study we did not detect significant antigenic variations between genotype C4 and B5 using 33 post-infection children sera, which is similar to other studies conducted in Taiwan [27,28]. Since the sample size for measuring cross-reactive neutralizing antibody titers in our study is small, it would be desirable to collect more sera from young children infected with different EV-A71 genotypes to measure cross-reactive neutralizing antibody titers. Moreover, vaccine candidates using different EV-A71 genotypes are evaluated in clinical trials in different countries (B2 in Singapore, B4 in Taiwan, and C4a in China). It is also critical to monitor cross-reactive neutralizing antibody responses using post-immunization sera collected from young children.

It is hard to compare different serological studies using different serological assays and sampling methods. The first international standard sera for measuring EV-A71 neutralizing antibody titers has been established [29]. It would be desirable to collect sera from different countries using similar sampling methods to measure cross-reactive neutralizing antibody titers using similar neutralization assays incorporating the international standard serum and recent circulating EV-A71 viruses.

One limitation of this study should be noted. Our study was a hospital-based study instead of a community-based random survey. A community study with random sampling is the most valid method to have a representative sample for a seroprevalence study. However, the cost of conducting a community survey in a city without comprehensive house-hold registration system may be too high to be feasible. Alternatively, a hospital-based study by using residual serum is relatively cost-effective to estimate the seroprevalence in a population (Table 1 and S2 Table). Our study site, Children’s Hospital 1, is the largest public hospital providing healthcare to children in HCMC and other southern provinces of Vietnam, which could eliminate financial barriers to healthcare access and may have good representativeness to the population. We further compared the geographical distribution of study participants and all non-HFMD/herpangina inpatients of CH1 during the study period. Among the study participants, 65% (n = 359) were from provinces other than HCMC, which was comparable with that of the non-HFMD/herpangina inpatients (58.3%). In addition, the population in provinces other than HCMC accounted for 69% of total population in southern Vietnam [30] (Table 2). Based on these comparisons, the sample representativeness of this study could be supported.

**Table 2:** Geographical distribution of study participants, all non-HFMD/herpangina inpatients of CH1, 2012–2013, and population in southern Vietnam.

Geographical area	Population in southern Vietnam [30] N (%)	Non-HFMD/herpangina inpatients in CH1 N (%)	Participants in this study N (%)
HCMC	12,300,000 (30.7%)	73,863 (41.7%)	194 (35.1%)
Provinces	27,817,703 (69.3%)	103,290 (58.3%)	359 (64.9%)
Total	40,117,703 (100.0%)	177,153 (100.0%)	553 (100.0%)

It is critical to understand EV-A71 disease burden for formulating national vaccination policy. Currently, national studies estimating EV-A71-related disease burden have been conducted in China [31] and Taiwan [32]. A similar study was recently conducted in southern Vietnam and found that a total cost of more than 90 million USD was caused by HFMD during 2016–2017, and the cost attributed to EV-A71 was much higher than that caused by other EV serotypes [33]. Moreover, harmonized laboratory-based enterovirus surveillance system is not available globally and it is urgently needed in Asian countries experiencing large-scale EV-A71 epidemics.

## Supporting information

S1 Table Age-specific seroprevalence of EV-A71 neutralizing antibodies by gender in Vietnamese children. (DOCX)

S2 Table Other EV-A71 seroprevalence studies conducted in Asian countries. (DOCX)
