# Supplementary material for: Seroprevalence of EV-A71 neutralizing antibodies following the 2011 epidemic in HCMC, Vietnam
Source: PLoS Negl Trop Dis. 2020 Mar 3;14(3):e0008124. doi: 10.1371/journal.pntd.0008124 (PMC7077839; doi:10.1371/journal.pntd.0008124)
Supplement: S1 Table — (DOCX) [file pntd.0008124.s001.docx]

Supplemental Table 1. Age-specific seroprevalence of EV-A71 neutralizing antibodies by gender in Vietnamese children

| Age (months) | Seroprevalence | | p-value |
| --- | --- | --- | --- |
|  | Male | Female |  |
| <6 | 16.1% | 13.3% | 0.805 |
| 6-11 | 15.0% | 22.2 | 0.634 |
| 12-23 | 24.1% | 23.8% | 0.979 |
| 24-35 | 32.4% | 23.5% | 0.514 |
| 36-47 | 58.3% | 59.1% | 0.955 |
| 48-59 | 58.3% | 70.6% | 0.390 |
| 60-71 | 65.0% | 68.4% | 0.795 |
| >=72 | 74.4% | 80.5% | 0.310 |
