# Supplementary material for: Seroprevalence of EV-A71 neutralizing antibodies following the 2011 epidemic in HCMC, Vietnam
Source: PLoS Negl Trop Dis. 2020 Mar 3;14(3):e0008124. doi: 10.1371/journal.pntd.0008124 (PMC7077839; doi:10.1371/journal.pntd.0008124)
Supplement: S2 Table — (DOCX) [file pntd.0008124.s002.docx]

Supplemental. Table 2. Other EV-A71 seroprevalence studies conducted in Asian countries

|  | Vietnam,  2006-07 [22] | Taiwan,  1999 [23] | Guangzhou, China, 2014-2015 [24] | Singapore,  1996-97 [25] |
| --- | --- | --- | --- | --- |
| Serum collection | Suspected dengue | Healthy children in community | Healthy children in community | Healthy children in hospital |
| Age-specific seroprevalence | 4%(6-11m)  9.5%(12-23m)  20.9%(24-35m)  84% (5-15 y) | 8-30% (12-23m)  11-36% (24-35m)  34-49% (36-47m)  56-58% (6-11y) | 35.3% (<2y)  43.3% (2-3y)  65.0% (3-4y)  76.7% (4-5y) | 1% (12-23m)  10% (24-35m)  20% (36-47m)  30% (48-59m)  50% (60-71m) |
| Median (years) | 4-5 | 5-6 | 3.5 | 5.5 |
